# Reduced risk of breast cancer associated with recreational physical activity varies by HER2 status

**DOI:** 10.1002/cam4.465

**Published:** 2015-04-27

**Authors:** Huiyan Ma, Xinxin Xu, Giske Ursin, Michael S Simon, Polly A Marchbanks, Kathleen E Malone, Yani Lu, Jill A McDonald, Suzanne G Folger, Linda K Weiss, Jane Sullivan-Halley, Dennis M Deapen, Michael F Press, Leslie Bernstein

**Affiliations:** 1 Division of Cancer Etiology, Department of Population Sciences, Beckman Research Institute, City of HopeDuarte, California, 91010; 2 Cancer Registry of NorwayPB 5313 Majorstuen, 0304, Oslo, Norway; 3 Department of Nutrition, Institute of Basic Medical Sciences, University of OsloOslo, Norway; 4 Department of Oncology, Karmanos Cancer Institute, Wayne State UniversityDetroit, Michigan, 48201; 5 Division of Reproductive Health, National Center for Chronic Disease Prevention and Health Promotion, Centers for Disease Control and PreventionAtlanta, Georgia, 30333; 6 Division of Public Health Sciences, Fred Hutchinson Cancer Research CenterSeattle, Washington, 98109; 7 College of Health and Social Services, New Mexico State UniversityLas Cruces, New Mexico, 88003; 8 Cancer Centers Branch, National Cancer InstituteBethesda, Maryland, 20850; 9 Department of Preventive Medicine, University of Southern CaliforniaLos Angeles, California, 90033; 10 Department of Pathology, Keck School of Medicine, University of Southern CaliforniaLos Angeles, California, 90033

**Keywords:** Breast cancer, ER, HER2, luminal A breast cancer, p53, physical activity, PR, Triple-negative breast cancer

## Abstract

Convincing epidemiologic evidence indicates that physical activity is inversely associated with breast cancer risk. Whether this association varies by the tumor protein expression status of the estrogen receptor (ER), progesterone receptor (PR), human epidermal growth factor receptor 2 (HER2), or p53 is unclear. We evaluated the effects of recreational physical activity on risk of invasive breast cancer classified by the four biomarkers, fitting multivariable unconditional logistic regression models to data from 1195 case and 2012 control participants in the population-based Women’s Contraceptive and Reproductive Experiences Study. Self-reported recreational physical activity at different life periods was measured as average annual metabolic equivalents of energy expenditure [MET]-hours per week. Our biomarker-specific analyses showed that lifetime recreational physical activity was negatively associated with the risks of ER-positive (ER+) and of HER2-negative (HER2−) subtypes (both *P*_trend_ ≤ 0.04), but not with other subtypes (all *P*_trend_ > 0.10). Analyses using combinations of biomarkers indicated that risk of invasive breast cancer varied only by HER2 status. Risk of HER2–breast cancer decreased with increasing number of MET-hours of recreational physical activity in each specific life period examined, although some trend tests were only marginally statistically significant (all *P*_trend_ ≤ 0.06). The test for homogeneity of trends (HER2– vs. HER2+ ) reached statistical significance only when evaluating physical activity during the first 10 years after menarche (*P*_homogeneity_ = 0.03). Our data suggest that physical activity reduces risk of invasive breast cancers that lack HER2 overexpression, increasing our understanding of the biological mechanisms by which physical activity acts.

## Introduction

Convincing epidemiologic evidence indicates that physical activity is inversely associated with breast cancer risk with an average decrease in risk of 25–30% for women in the highest versus the lowest category of physical activity 1. Although previous studies have examined whether the physical activity-breast cancer association varies by the tumor protein expression status of the estrogen receptor (ER) and the progesterone receptor (PR) 2–23, little is known as to whether the association varies by the human epidermal growth factor receptor 2 (HER2) or p53.

Several biological mechanisms have been proposed that may account for the association between physical activity and breast cancer 24–29. Physical activity may reduce a woman’s cumulative exposure to biologically available estrogens by delaying the onset of menarche, reducing the number of ovulatory cycles she experiences, or decreasing body fat, which would decrease the aromatization of androgens thereby reducing estrogens levels 30. Estrogens are mitogens in breast tissue, stimulating mammary cell proliferation which increases the chance of random genetic mutations through ER activation 31,32. Due to the possible involvement of hormone-related mechanisms, the associations between physical activity and breast cancer have been evaluated by ER status, or ER and PR status jointly 2–23. The majority of these studies found that physical activity was associated with a lower risk of breast cancer regardless of ER or ER/PR 7–21. Five studies observed a statistically significant association of physical activity with lower breast cancer risk only among women with ER-positive (ER+) or ER+ plus PR-positive (PR+) 2–6. In two studies, physical activity was associated with a reduction in breast cancer risk only for women with ER-negative (ER−) breast cancer 22,23.

Gene expression studies with cDNA microarray technology have demonstrated that triple negative (TN, ER−/PR− as well as human epidermal HER2-negative, HER2−) breast cancers are often characterized by a basal-like molecular profile, which exhibits overexpression of a number of genes involved in cell proliferation and differentiation, p-21 mediated pathway, and G1-S checkpoints of cell cycle signaling pathways; whereas ER/PR+/HER2− and ER/PR+/HER2+ are often characterized by luminal molecular profiles, which are associated with the ER signaling pathway 33,34. Note that we use the notation “ER/PR+” to represent “ER+ and/or PR+” throughout this article. Owing to the different pathways identified for different breast cancer subtypes, the inverse association of physical activity with breast cancer could vary by subtype defined by ER, PR, and HER2 status. The findings from three epidemiologic studies on this topic are mixed 3,35,36.

Little is known about p53 status and the physical activity-breast cancer association. Among mice with a single defective *p53* allele, treadmill running exercise was associated with an increased rate of mammary tumor development 37. Yet, no epidemiologic data have been published prior to 2015 regarding this association.

We previously reported that risk of both ER+ and ER− invasive breast cancer decreased with increasing levels of recreational physical activity using data from the five study sites of the Women’s Contraceptive and Reproductive Experiences (CARE) Study 10. In a sub-study conducted at two of the participating Women’s CARE study sites, where tumor tissue was collected, we showed that hormone-related risk factors (e.g., number of full-term pregnancies and age at first full-term pregnancy) were associated with the risk of ER/PR+/HER2− breast cancer, but not with TN breast cancer 38. Here we examine whether the benefits of physical activity vary by the tumor protein expression status of ER, PR, HER2, and p53 in order to provide greater insight into biological mechanisms underlying the association between physical activity and risk of breast cancer.

## Materials and Methods

### Study population and data collection

The participants for this analysis include women from Detroit and Los Angeles (LA), two of the five participating sites (Atlanta, Detroit, LA, Philadelphia, Seattle) in the Women’s CARE Study 39. The Women’s CARE Study, which was supported by National Institute of Child Health and Human Development (NICHD), was a population-based, case–control study designed to examine risk factors for invasive breast cancer among US-born white women and black women 39. The age distribution and participant response rates by study site, case–control status and race have been published 39. Tissue collection, as part of the Women’s CARE Study, was supported by NICHD for the Detroit and LA study sites, as advised by the Women’s CARE Steering Committee 39.

Case participants in the Women’s CARE Study had no prior diagnosis of invasive or *in situ* breast cancer and were diagnosed with their first primary invasive breast cancer (International Classification of Diseases for Oncology codes C50.0–C50.9) between July 1994 and April 1998. Control participants were women with no history of invasive or *in situ* breast cancer who were identified by random digit dialing. Control participants were frequency matched to the expected distribution of cases in strata defined by 5 year age groups, ethnicity (white or black), and residence located in the same geographic (study) region. The Women’s CARE Study recruited 1921 case and 2034 control participants from Detroit and LA. The interview response rates were 74.7% for cases in Detroit, 74.1% for controls in Detroit, 73.3% for cases in LA, and 73.7% for controls in LA. All participants for this study provided written informed consent and the study protocol was approved by the Institutional Review Boards at the University of Southern California (USC), the Karmanos Cancer Institute Center, the Centers for Disease Control and Prevention, and the City of Hope.

The Women’s CARE Study collected demographic characteristics, detailed information about current and past recreational physical activity, menstrual and reproductive history, family history of breast cancer, body size measures including height and weight, history of oral contraceptive use, and information pertaining to other factors from each participant during an in-person interview conducted from August 1994 through December 1998. Information was recorded up to a predetermined reference date for each participant. The reference date was the date of diagnosis for women with breast cancer or the date of the initial telephone screening of the household for control participants.

### Measures of recreational physical activity

Details regarding the measures of recreational physical activity in the Women’s CARE Study have been published elsewhere 10. Briefly, the Women’s CARE Study documented all episodes of exercise activity in which a participant engaged throughout her lifetime up to her reference date, and recorded details of activities in chronologic order starting with the first activity recalled by the respondent. The information collected for each activity episode included the type of activity, the age at which the woman started and stopped the activity, the number of months per year of participation in the activity, and average duration in hours per week. The activities reported included any organized sports activities, such as school sports or teams, and individual activities, such as walking, jogging, running, hiking, bicycling, aerobics, swimming, and dancing. The details as to the extent of the activity were also recorded. For example, for swimming, we collected the types of swimming including recreational swimming, snorkeling, swimming laps, or training for competitive swimming.

The average number of hours of exercise activity per week for each year of age for each participant was estimated. Women were considered to be inactive at any given age if they reported no activity for that age or if their average number of hours per week of activity for that age was less than 0.67 h (i.e., equivalent to less than 2 h/week for 4 months). The metabolic equivalents of energy expenditure (MET)-hours per week for each age were estimated by multiplying together the number of hours per week a woman spent in a particular activity, the proportion of the year spent in that activity, and the estimated MET score for the activity based on the Compendium of Physical Activity 40. A measure of lifetime activity was defined as average annual exercise activity from age 10 years to the woman’s age on her reference date in hours per week and in MET-hours per week. The average number of MET-hours per week was also assessed for the following specific times: the first 10 years after menarche, ages 10–19 years, ages 20–34 years, and the 10 years before each woman’s reference date.

### Assessment of biomarkers

Paraffin-embedded tumor blocks were obtained from pathology laboratories where diagnoses were made for 1333 participating breast cancer cases (Detroit: 414, LA: 919). Approximately 80% of the blocks requested were received. Tumor blocks were carefully reviewed and processed in the centralized pathology laboratory of Dr. Michael F. Press at USC.

We excluded 113 tumor samples because the tumor blocks contained either no tumor tissue (*n* = 46), insufficient tissue for the laboratory assays (*n* = 3), only carcinoma *in situ* (*n* = 56), or only hematoxylin and eosin-stained tissue sections (*n* = 8); we also excluded 14 samples that had other problems that made evaluation of the tumor ER, PR, HER2, or p53 difficult. Expression of ER, PR, HER2, and p53 was determined for 1206 samples (Detroit: 367, LA: 839).

ER and PR expression was determined using previously published immunohistochemistry (IHC) methods 41,42. Immunostaining results for ER and PR expression were interpreted in a blind fashion and scored semi-quantitatively on the basis of the visually estimated percentage of positively stained tumor cell nuclei. At least 100 tumor cells were examined for each specimen and samples with ≥1% of immunostained tumor cell nuclei were considered positive for ER and PR 43.

HER2 expression was determined by IHC using the 10H8 monoclonal antibody 44 to assess HER2 membrane protein immunostaining. Immunostaining results for HER2 were categorized as no (0) or weak (1+), moderate (2+), and strong (3+) membrane immunostaining. No (0) or weak (1+) membrane immunostaining was classified as low HER2 expression (HER2−) whereas moderate (2+) or strong membrane immunostaining (3+) was classified as HER2 overexpression (HER2+). This was based on previous validation results from the same pathology laboratory, indicating that the agreement between 10H8-IHC and fluorescent *in situ* hybridization (FISH) analysis was 92%; discordant results were found for 5.7% of tumor samples, which scored as 0 or 1+ by 10H8-IHC, but showed *HER2* gene amplification; and 2.1% of tumor samples, which scored as 2+ or 3+ by 10H8-IHC, but showed no *HER2* gene amplification 44.

The expression of p53 protein was determined by IHC using the monoclonal mouse antibody DO7 (Oncogene Science, Inc. Cambridge, MA) and BP 53-12-1 (Biogenex, San Ramon, CA) to measure p53 nuclear protein immunostaining. Based on findings from previous studies, comparing p53 mutations in exons 2–11 with p53 protein expression levels 45,46, ≥10% nuclear staining for p53 protein was deemed positive 47.

### Statistical analyses

We used Pearson Chi-squared tests to compare frequency distributions of categorical variables. Because of the non-normal distributions of age at reference date and body mass index (BMI) 5 years before the reference date, we conducted the nonparametric Wilcoxon test to evaluate differences in these two variables between case participants and control participants.

For case–control comparisons, we fit multivariable polychotomous unconditional logistic regression models 48 to data to estimate the odds ratios (ORs) and corresponding 95% confidence intervals (CIs) of breast cancer associated with lifetime recreational physical activity (1) by the expression status of each individual receptor for all women, premenopausal women, and postmenopausal women, (2) by various combinations of ER, PR and HER2 status including two common subtypes (TN and ER/PR+/HER2−), which were further stratified by p53 status, and (3) by three levels of HER2 expression (none/weak, moderate, strong expression). We also examined the association between time-period-specific or age-specific recreational physical activity and breast cancer risk according to HER2 status. Moreover, since differential recall of detailed physical activity history between cases and controls might occur, we conducted case–case comparisons for ER− versus ER+, PR− versus PR+, HER2− versus HER2+, and p53− versus p53+ patients using a multivariable unconditional logistic regression approach 48.

We used previously published categories of average MET-hours per week of physical activity (less than or equal to 2.2, 2.3 to 6.6, 6.7 to 15.1, or at least 15.2 annual MET hour/week), which were generated according to approximate quartiles of the distribution of all Women’s CARE Study control participants classified as active 10. We included the following factors, selected a priori, as potential confounders in all multivariable logistic regression models: study site (Detroit or LA), race (white or black), education (high school graduate or a lower level of education, attended technical school or college, but did not graduate, or college graduate), age (in 5 year age groups from 35–39 to 60–64), family history of breast cancer [first-degree (mother, sister, or daughter); no first-degree family history including 4% of participants with uncertain answers], age at menarche (less than or equal to 11, 12, 13, or at least 14 years), parity (nulligravid, pregnant but no full-term pregnancy, or parity 1, 2, 3, or 4+), a four-category variable combining menopausal status and hormone therapy (HT) use (premenopausal, postmenopausal and never HT use, postmenopausal and ever HT use, or unknown menopausal status), BMI five years before the reference date (continuous variable, kg/m^2^), duration of OC use (never, less than 1 year, 1–4 years, 5–9 years, or at least 10 years). When we examined the association between time-period-specific or age-specific recreational physical activity and breast cancer risk, we did not mutually adjust time-period-specific and age-specific physical activity as some of the periods overlap and, in addition, physical activity measures are highly correlated (e.g., the Spearman correlation coefficient for average-annual MET-hours/wk of physical activity at ages 10–19 years and 20–34 years was 0.69); we included women who engaged in recreational physical activity only in other time periods or other age groups as a separate category.

Tests for trend were conducted by fitting ordinal values corresponding to categories of recreational physical activity in our models and testing whether the coefficient (slope of the dose response) differed from zero. When conducting tests for trend for time-period-specific or age-specific physical activity variables, we excluded women who engaged in recreational physical activity only in other time periods or other age groups. We also conducted Wald chi-square tests for homogeneity of the associations with recreational physical activity across different subtypes of breast cancer by fitting a model using ordinal values.

We excluded 11 case participants and 22 control participants with missing information on physical activity (2 cases, 3 controls), parity (1 case, 4 controls), BMI (4 cases, 9 controls), or OC use (4 cases, 6 controls). This resulted in 1195 cases (581 premenopausal, 497 postmenopausal, and 117 with unknown menopausal status) and 2012 controls available for the current analysis (929 premenopausal, 831 postmenopausal, and 252 with unknown menopausal status). Among 1328 postmenopausal women, 827 women (307 cases, 520 controls) reported having ever used HT.

When reporting the results of univariate comparisons between case participants and control participants, trend tests, or homogeneity tests, we considered a two-sided *P* value less than 0.05 as statistically significant. We did not adjust *P* values for multiple comparisons as these analyses were considered as exploratory 49. All analyses were performed using the SAS statistical package (Version 9.2, SAS Institute, Cary, NC).

## Results

### Characteristics of cases and controls

Overall, case participants were more likely than control participants to be better educated (*P*_*χ2*_ = 0.01), to have a first-degree breast cancer family history (*P*_*χ2*_ < 0.0001), and to never have been pregnant (*P*_*χ2*_ = 0.02) (Table1). The case–control differences in education and pregnancy history were restricted to LA women, whereas the difference in first-degree breast cancer family history was observed for both LA and Detroit women.

**Table 1:** Characteristics of invasive breast cancer patients and control participants from Detroit and Los Angeles components of the Women’s CARE Study

	Overall			Detroit			Los Angeles		
	Controls (*n* = 2012)	Cases (*n* = 1195)	*P*-value^1^	Controls (*n* = 771)	Cases(*n* = 361)	*P*-value^1^	Controls (*n* = 1241)	Cases (*n* = 834)	*P*-value^1^
Race									
White	57.1%	56.6%	0.77	57.7%	62.1%	0.17	56.7%	54.2%	0.26
Black	42.9%	43.4%		42.3%	38.0%		43.3%	45.8%	
Education									
≤High school	40.0%	35.9%	0.01	46.2%	44.9%	0.54	36.2%	32.0%	0.05
Technical school or some college	33.7%	38.7%		30.1%	33.2%		35.9%	41.0%	
College graduate	26.3%	25.4%		23.7%	21.9%		27.9%	27.0%	
Mean age at reference date (SD), years	48.9 (8.4)	49.0 (8.6)	0.67^2^	49.0 (8.5)	48.7 (8.8)	0.56^2^	48.8 (8.4)	49.1 (8.5)	0.35^2^
First-degree breast cancer family history	8.2%	15.6%	*<*0.0001	9.3%	17.2%	0.0001	7.5%	14.9%	*<*0.0001
Age at menarche, years									
≤11	28.5%	25.6%	0.17	29.3%	26.6%	0.70	28.0%	25.2%	0.31
12	25.9%	27.2%		25.4%	26.6%		26.2%	27.5%	
13	25.6%	28.2%		26.1%	28.5%		25.3%	28.1%	
≥14	20.0%	19.0%		19.2%	18.3%		20.6%	19.3%	
Number of full-term (>26 week) pregnancies									
Never pregnant	8.8%	11.2%	0.02	8.3%	10.0%	0.91	9.0%	11.8%	0.02
Only non-full-term pregnancy	7.9%	7.2%		5.1%	5.3%		9.6%	8.0%	
1	15.6%	17.9%		15.7%	17.2%		15.6%	18.2%	
2	28.5%	29.6%		29.4%	28.0%		28.0%	30.3%	
3	19.4%	17.3%		19.8%	19.1%		19.1%	16.6%	
≥4	19.9%	16.7%		21.7%	20.5%		18.8%	15.1%	
Menopausal status									
Premenopausal	46.2%	48.6%	0.12	46.0%	49.3%	0.12	46.3%	48.3%	0.21
Postmenopausal									
Never HT use	15.5%	15.9%		17.6%	21.3%		14.1%	13.6%	
Ever HT use	25.8%	25.7%	24.1%	19.7%	26.9%	28.3%			
Unknown	12.5%	9.8%	12.2%	9.7%	12.7%	9.8%			
Mean body mass index 5 years before reference date (SD), years	26.1 (6.0)	26.0 (5.8)	0.88^2^	26.2 (6.1)	26.1 (6.0)	0.66^2^	26.0 (6.0)	26.0 (5.7)	0.84^2^
Duration of oral contraceptive use, years									
Never	20.3%	21.2%	0.84	17.9%	23.3%	0.18	21.8%	20.3%	0.78
<1	17.7%	17.7%		16.6%	15.2%		18.5%	18.8%	
1–4	26.8%	26.6%		28.4%	29.9%		25.9%	25.2%	
5–9	19.7%	18.2%		21.7%	18.0%		18.5%	18.4%	
≥10	15.5%	16.2%		15.4%	13.6%		15.5%	17.4%	
ER									
Negative		42.0%			44.0%			41.1%	
Positive		58.0%			56.0%			58.9%	
PR									
Negative		44.6%			46.5%			43.8%	
Positive		55.4%			53.5%			56.2%	
HER2									
Negative		81.9%			84.8%			80.7%	
Positive		18.1%			15.2%			19.3%	
P53									
Negative		72.1%			80.6%			68.4%	
Positive		27.9%			19.4%			31.6%	

SD, standard deviation.

1 *P*-value ascertained from Pearson *χ*^2^ test, except where otherwise noted.

2 *P*-value from nonparametric Wilcoxon tests.

### Associations of breast cancer defined by the status of individual receptors with lifetime recreational physical activity

As previously reported among all participants of the Women’s CARE Study 50, lifetime recreational physical activity was associated with a decreased risk of ER− and ER+ breast cancer, but only the result for ER+ disease was statistically significant in our sample of LA and Detroit women (*P*_trend_ = 0.67 for ER− vs. *P*_trend_ = 0.03 for ER+, Table2). Analyses by HER2 status showed that the ORs of HER2− breast cancer declined with increasing lifetime MET-hours of physical activity (*P*_trend_ = 0.04), whereas no trend was observed for HER2+ breast cancer (*P*_trend_ = 0.93). Homogeneity tests of trends neither between ER− and ER+ nor between HER2− vs. HER2+ was statistically significant (both *P*_homogeneity of trends_ ≥ 0.19). Our data showed no evidence of an association between recreational physical activity and breast cancer risk that varied according to PR or p53 protein status. Although HER2− cases did not differ statistically from HER2+, HER2− cases were less likely to have engaged in recreational physical activity. Moreover, the results observed for all participants are likely driven by those of premenopausal women since we did not observe any association among postmenopausal women (Table S1).

**Table 2:** Multivariable adjusted^1^ OR and 95% CI for invasive breast cancer defined by the status of each individual receptor with lifetime recreational physical activity

Average exercise activity (annual MET hour/week)	No. of participants			OR (95% CI)		
	Controls	Receptor negative cases	Receptor positive cases	Receptor negative cases vs. controls	Receptor positive cases vs. controls	Receptor negative vs. receptor positive
Cases sub-grouped by ER status						
Inactive	500	136	171	Referent	Referent	Referent
≤2.2	373	76	132	0.78 (0.57–1.07)	0.95 (0.72–1.25)	0.79 (0.54–1.15)
2.3–6.6	369	102	148	1.02 (0.75–1.38)	1.03 (0.78–1.35)	0.98 (0.68–1.40)
6.7–15.1	374	88	127	0.85 (0.62–1.17)	0.83 (0.63–1.10)	1.00 (0.68–1.47)
≥15.2	396	100	115	0.91 (0.67–1.24)	0.73 (0.55–0.98)	1.29 (0.88–1.89)
Trend *P*-value				0.67	0.03	0.14
Homogeneity of trends for case–control comparison				*P *= 0.19		
Cases sub-grouped by PR status						
Inactive	500	148	159	Referent	Referent	Referent
≤2.2	373	91	117	0.87 (0.65–1.18)	0.88 (0.67–1.17)	0.95 (0.65–1.37)
2.3–6.6	369	107	143	1.00 (0.74–1.34)	1.04 (0.79–1.38)	0.92 (0.65–1.31)
6.7–15.1	374	90	125	0.80 (0.59–1.10)	0.86 (0.65–1.15)	0.91 (0.62–1.32)
≥15.2	396	97	118	0.81 (0.60–1.10)	0.81 (0.60–1.08)	1.02 (0.70–1.48)
Trend *P*-value				0.15	0.17	0.94
Homogeneity of trends for case–control comparison				*P* = 0.91		
Cases sub-grouped by HER2 status						
Inactive	500	258	49	Referent	Referent	Referent
≤2.2	373	169	39	0.83 (0.65–1.06)	1.14 (0.72–1.78)	0.75 (0.46-1.21)
2.3–6.6	369	201	49	0.96 (0.75–1.21)	1.38 (0.89–2.13)	0.73 (0.46-1.16)
6.7–15.1	374	173	42	0.77 (0.60–0.99)	1.18 (0.75–1.87)	0.61 (0.38-1.00)
≥15.2	396	178	37	0.77 (0.60–0.99)	0.98 (0.61–1.58)	0.75 (0.45-1.24)
Trend *P*-value				0.04	0.93	0.14
Homogeneity of trends for case–control comparison				*P* = 0.23		
Cases sub-grouped by p53 status						
Inactive	500	213	94	Referent	Referent	Referent
≤2.2	373	149	59	0.88 (0.68–1.14)	0.89 (0.62–1.27)	1.03 (0.68–1.54)
2.3–6.6	369	185	65	1.08 (0.84–1.39)	0.91 (0.63–1.30)	1.21 (0.82–1.80)
6.7–15.1	374	164	51	0.91 (0.70–1.18)	0.67 (0.46–0.98)	1.43 (0.93–2.19)
≥15.2	396	150	65	0.81 (0.62–1.06)	0.81 (0.56–1.16)	0.96 (0.64–1.46)
Trend *P*-value				0.20	0.11	0.59
Homogeneity of trends for case–control comparison				*P = *0.50		

OR, odds ratio; CI, confidence interval; ER, estrogen receptor; PR, progesterone receptor; HER2, human epidermal growth factor receptor 2.

1 Adjusted for study site, race, education, age, family history of breast cancer, age at menarche, parity, a four-category variable combining menopausal status and hormone therapy use, body mass index, and the duration of oral contraceptive use.

### Associations of breast cancer defined by combinations of biomarkers with lifetime recreational physical activity

Analyses by the status of ER and HER2 jointly showed that lifetime MET-hours of physical activity were associated with decreased risks for the HER2− subtypes (ER−/HER2− and ER+/HER2− breast cancers), but only the result for ER+/HER2− was statistically significant (Table3, *P*_trend_ = 0.01 for ER+/HER2−). Our data did not provide any evidence that lifetime MET-hours of physical activity was associated with a reduced risk for HER2+ subtypes including ER−/HER2+ and ER+/HER2+ breast cancer (both *P*_trend_ ≥ 0.88). However, we found no difference in the trends across subtypes defined by ER and HER2 (test for homogeneity of trends: *P* = 0.38).

**Table 3:** Multivariable adjusted^1^ OR and 95% CI for invasive breast cancer defined by combinations of biomarkers with lifetime recreational physical activity

		Cases sub-grouped by ER/HER2							
Average exercise activity (annual MET h/week)	No. of controls	No. of cases	OR (95% CI)	No of cases	OR (95% CI)	No. of cases	OR (95% CI)	No. of cases	OR (95% CI)
Cases sub-grouped by ER/HER2 status									
		**ER−/HER2−**		**ER−/HER2+**		**ER+/HER2−**		**ER+/HER2+**	
Inactive	500	106	Referent	30	Referent	152	Referent	19	Referent
≤2.2	373	59	0.76 (0.53–1.08)	17	0.86 (0.46–1.60)	110	0.88 (0.66–1.17)	22	1.52 (0.80–2.89)
2.3–6.6	369	78	0.97 (0.70–1.36)	24	1.18 (0.66–2.09)	123	0.95 (0.71–1.26)	25	1.67 (0.89–3.16)
6.7–15.1	374	66	0.77 (0.54–1.10)	22	1.15 (0.63–2.08)	107	0.78 (0.58–1.05)	20	1.27 (0.65–2.48)
≥15.2	396	81	0.91 (0.65–1.27)	19	0.92 (0.50–1.72)	97	0.69 (0.51–0.94)	18	1.09 (0.54–2.19)
Trend *P*-value			0.57		0.88		0.01		0.99
Homogeneity of trends			*P = *0.38						

		Cases sub-grouped by ER/PR/HER2							
Average exercise activity (annual MET h/week)	No. of controls	No. of cases	OR (95% CI)	No of cases	OR (95% CI)	No. of cases	OR (95% CI)	No. of cases	OR (95% CI)
Cases sub-grouped by ER/PR/HER2 status									
		**TN**		**HER2-enriched**		**ER/PR+/HER2−**		**ER/PR+/HER2+**	
Inactive	500	93	Referent	25	Referent	165	Referent	24	Referent
≤2.2	373	52	0.78 (0.54–1.13)	17	1.07 (0.56–2.03)	117	0.86 (0.65–1.14)	22	1.20 (0.65–2.20)
2.3–6.6	369	67	0.98 (0.68–1.39)	20	1.20 (0.64–2.24)	134	0.95 (0.72–1.25)	29	1.54 (0.86–2.76)
6.7–15.1	374	52	0.70 (0.48–1.03)	19	1.21 (0.64–2.30)	121	0.81 (0.61–1.08)	23	1.17 (0.63–2.17)
≥15.2	396	71	0.92 (0.64–1.31)	15	0.88 (0.44–1.74)	107	0.70 (0.52–0.94)	22	1.08 (0.58–2.02)
Trend *P*-value			0.48		0.94		0.02		0.84
Homogeneity of trends			*P *= 0.52						

OR, odds ratio; CI, confidence interval; ER, estrogen receptor; PR, progesterone receptor; HER2, human epidermal growth factor receptor 2; TN, triple negative. TN, ER−/PR−/HER2−; HER2-enriched, ER−/PR−/HER2+; ER/PR+/HER2−, ER+ or PR+ plus HER2−; ER/PR+/HER2+, ER+ or PR+ plus HER2+.

1 Adjusted for study site, race, education, age, family history of breast cancer, age at menarche, parity, a four-category variable combining menopausal status and hormone therapy use, body mass index, and the duration of oral contraceptive use.

Analyses combining ER, PR, and HER2 also demonstrated that lifetime MET-hours of physical activity were inversely associated with the risk for HER2− subtypes, especially for ER/PR+/HER2− subtype (*P*_trend_ = 0.02), but were not associated with HER2+ subtypes (HER2-enriched, ER/PR+/HER2+; both *P*_trend_ ≥ 0.84). The difference in trends across the four subtypes defined by ER/PR/HER2 was not statistically significant (test for homogeneity of trends: *P *=* *0.52). Subclassification of two common subtypes (TN and ER/PR+/HER2−) by p53 status did not further differentiate the associations of these subtypes with recreational physical activity (test for homogeneity of trends: *P *=* *0.44, results not shown). We assessed whether lifetime recreational physical activity was associated with breast cancer with each level of HER2 expression (negative/weakly positive, moderately positive, strongly positive; Table S2). This analysis showed that the ORs of HER2− breast cancer declined with increasing lifetime MET-hours of physical activity (*P*_trend_ = 0.04), but no association was observed for breast cancers that were either moderate or strong expressers of HER2 (both *P*_trend_ ≥ 0.76).

### Associations of breast cancer defined by HER2 status with recreational physical activity in which a woman engaged during specific time periods or age periods

HER2–breast cancer was inversely associated with the MET-hours of physical activity for each specific time period of life that we examined, although some tests for trend were only marginally statistically significant (Table4, all *P*_trend_ ≤ 0.06). No associations were found for HER2+ breast cancers (all *P*_trend_ ≥ 0.25). The difference in trends between HER2− and HER2+ breast cancer was statistically significant for recreational physical activity only in the first 10 years after menarche (test for homogeneity of trends: *P *=* *0.03) and was marginally statistically significant for physical activity in which the woman engaged at ages 10–19 years (test for homogeneity of trends: *P *=* *0.06), whereas the trends in risk did not differ statistically for activity at ages 20–34 or 10 years before reference date (both tests for homogeneity of trends: *P *≥* *0.12).

**Table 4:** Multivariable adjusted^1^ OR and 95% CI for invasive breast cancer defined by HER2 status with recreational physical activity in specific time periods or age periods

Average exercise activity (annual MET h/week)	No. of participants			OR (95% CI)		
	Controls	HER2− cases	HER2+ cases	HER2− cases versus controls	HER2+ cases versus controls	HER2− versus HER2+
First 10 years after menarche						
Inactive^2^	504	260	49	Referent	Referent	Referent
≤2.2	97	44	5	0.82 (0.55–1.22)	0.54 (0.21–1.42)	1.54 (0.57–4.19)
2.3–6.6	186	94	30	0.85 (0.63–1.15)	1.69 (1.02–2.80)	0.55 (0.32–0.94)
6.7–15.1	241	120	28	0.84 (0.64–1.11)	1.25 (0.75–2.08)	0.67 (0.39–1.16)
≥15.2	412	187	43	0.77 (0.60–0.98)	1.10 (0.70–1.74)	0.67 (0.41–1.08)
Trend *P*-value				0.05	0.25	0.02
Homogeneity of trends for case–control comparison				*P *= 0.03		
Exercise only in other time period(s)	572	274	61	0.87 (0.70–1.08)	1.17 (0.78–1.75)	0.74 (0.48–1.14)
Ages 10–19 years						
Inactive^3^	500	258	49	Referent	Referent	Referent
≤2.2	76	49	9	1.13 (0.75–1.68)	1.22 (0.57–2.64)	0.95 (0.42–2.12)
2.3–6.6	195	83	22	0.71 (0.52–0.97)	1.19 (0.68–2.07)	0.57 (0.31–1.02)
6.7–15.1	221	109	19	0.80 (0.60–1.07)	0.87 (0.49–1.55)	0.91 (0.50–1.66)
≥15.2	409	187	45	0.78 (0.61–1.00)	1.14 (0.73–1.80)	0.67 (0.41–1.08)
Trend *P*-value				0.02	0.53	0.05
Homogeneity of trends for case–control comparison				*P* = 0.06		
Exercise only in other age group(s)	611	293	72	0.87 (0.71–1.08)	1.27 (0.85–1.87)	0.70 (0.46–1.06)
Ages 20–34 years						
Inactive^3^	500	258	49	Referent	Referent	Referent
≤2.2	164	76	10	0.82 (0.59–1.13)	0.63 (0.31–1.29)	1.43 (0.67–3.04)
2.3–6.6	225	131	33	1.03 (0.78–1.36)	1.54 (0.95–2.52)	0.66 (0.39–1.11)
6.7–15.1	286	148	29	0.89 (0.68–1.16)	1.02 (0.61–1.69)	0.83 (0.49–1.42)
≥15.2	365	151	33	0.72 (0.56–0.93)	0.92 (0.57–1.50)	0.76 (0.45–1.27)
Trend *P*-value				0.03	0.85	0.11
Homogeneity of trends for case–control comparison				*P *= 0.20		
Exercise only in other age group(s)	472	215	62	0.81 (0.64-1.02)	1.44 (0.95-2.17)	0.56 (0.36-0.87)
10 years before reference date						
Inactive^3^	500	258	49	Referent	Referent	Referent
≤2.2	188	90	19	0.89 (0.66–1.21)	1.06 (0.60–1.86)	0.89 (0.48–1.62)
2.3–6.6	277	140	32	0.88 (0.67–1.14)	1.26 (0.78–2.05)	0.70 (0.42–1.17)
6.7–15.1	333	172	42	0.90 (0.70–1.16)	1.31 (0.84–2.06)	0.69 (0.43–1.12)
≥15.2	441	200	45	0.78 (0.62–0.99)	1.09 (0.70–1.71)	0.67 (0.42–1.09)
Trend *P*-value				0.06	0.55	0.07
Homogeneity of trends for case–control comparison				*P* = 0.12		
Exercise only in other time period(s)	273	119	29	0.74 (0.56–0.98)	1.11 (0.67–1.83)	0.69 (0.40–1.17)

OR, odds ratio; CI, confidence interval. HER2, human epidermal growth factor receptor 2.

1 Adjusted for study site, race, education, age, family history of breast cancer, age at menarche, parity, a four-category variable combining menopausal status and hormone therapy use, body mass index, and the duration of oral contraceptive use.

2 Inactive between age at menarche and reference date.

3 Inactive between age 10 years and reference date.

When we compared HER2− cases with HER2+ cases, ORs decreased with increasing MET-hours of physical activity for all specific time periods. However, the association was statistically significant for physical activity in the first 10 years after menarche (*P*_trend_ = 0.02), but not for physical activity in other time periods (all *P*_trend_ ≥ 0.05).

## Discussion

Our analyses for tumor marker-specific breast cancer risk showed that lifetime recreational physical activity was only associated with a lower risk of ER+ and of HER2− breast cancer. Further analyses by the various combinations of ER, HER2, PR, and p53, revealed that the protective effect of lifetime recreational physical activity on breast cancer risk varied only by HER2 status.

The results of three previous studies that have examined the association between physical activity and breast cancer subtypes defined by ER, PR, and HER2 status are mixed 3,35,36. The Women’s Health Initiative (WHI) Cohort Study reported that the risk of ER+ and TN breast cancers were both inversely associated with baseline recreational physical activity (MET-hours/week), but no data were reported on whether HER2 status alone impacted the inverse association between recreational physical activity and breast cancer risk 36. Two case–control studies evaluated whether physical activity is associated with breast cancer risk according to HER2 status 3,35. In a population-based case–control study of postmenopausal women, leisure-time physical activity (MET-hours/week) after age 50 years was associated with lower risk of ER+/PR+ breast cancer, but not ER−/PR− breast cancer; risk did not vary further by HER2 status 3. In another population-based case–control study of women aged 20–54 years 35, women whose exercise activity in the year before interview was at or above the median level had a lower risk for all subtypes of breast cancer defined by ER/PR/HER2, except for the ER/PR+/HER2+ subtype. Our analyses showed that recreational physical activity was inversely associated with reduced risk for HER2− but not HER2+ breast cancer. Discrepancies in the results by study may be due to different time periods of physical activity assessed. These time periods included physical activity after age 50 years up to reference date (date of diagnosis for cases and date of interview for controls) 3 and physical activity only in the year before interview 35. In our study, a more comprehensive measure of physical activity was evaluated including physical activity over a lifetime plus four specific time periods of life. Moreover, the discrepancies in the results by study could also be due to use of different cut-points to define the status of HER expression or different methods to assay HER2. For example, one study defined HER2+ as tumors which were judged to be low/moderate or high intensity staining on IHC 35. In our study, we defined HER2+ as moderate or high intensity staining on IHC. Our data showed that recreational physical activity was not associated with breast cancers that were either moderate or strong expressers of HER2.

Moreover, the case–case comparison approach is a useful exploratory tool to examine etiologic heterogeneity between subtypes 51. Heterogeneity between subtypes may represent different etiologic mechanisms for the two groups of cases or it may represent a different strengths of effect operating through the same mechanism 51. One 35 of the three previous studies on this topic 3,35,36 reported case–case comparison data for the subtypes defined by ER/PR/HER2 (each subtype compared to ER/PR+/HER2−). Recreational physical activity for ER/PR+/HER2+ cases was more likely to be at or above the median level of activity than for ER/PR+/HER2− cases (OR = 1.73, 95% CI = 1.00–3.00) 35. In line with previous findings, our case–case comparisons showed that HER2− cases were less likely than HER2+ cases to have a higher annual MET hour/week of recreational physical activity, although the negative association was only statistically significant for physical activity in the first 10 years after menarche.

HER2, a transmembrane tyrosine kinase receptor protein, normally cooperates with three other HER receptors in various growth signaling pathways to regulate cell growth, differentiation, and survival 52. HER2 is overexpressed in approximately 15–25% of breast carcinoma specimens 53. The most common mechanism leading to HER2 overexpression is amplification of the *HER2* proto-oncogene 54,55 located on chromosome 17q21. Tumors that overexpress HER2 are more likely to grow rapidly, metastasize, and be resistant to endocrine therapy 56. Overexpression of HER2 may disrupt normal cell control mechanisms, potentially leading to the formation of aggressive tumor cells 57,58. Studies on stem/progenitor cells as initiators of breast cancer showed that HER2 overexpression increased the stem/progenitor cell populations of normal and malignant mammary cells. Increasing the stem/progenitor cell population may lead to tumorigenesis, tumor invasion, or metastasis 59. Although we have no explanation as to why the association between physical activity and breast cancer varied by HER2 status in our study, it is plausible that recreational physical activity may not exert a protective effect on breast cells if normal cell control mechanisms have been disrupted or if overexpression of HER2 has increased the stem/progenitor cell population. Further research will be needed to explore the possible mechanisms.

This study has several limitations. First, although recall error was minimized by assessing exercise activity in conjunction with the completion of a calendar of life events to facilitate recall and by recording activities at every age throughout life in the Women’s CARE Study, we cannot rule out the possibility that women’s history of activity was misclassified, especially for years in the distant past. This classification could differ between case participants and control participants, but it is unlikely to differ between HER2− and HER2+ case participants. Second, we did not request tissue for all eligible case participants due to funding constraints. We compared our measures of physical activity for eligible case participants with and without known ER, PR, HER2, and p53 status. No statistically significant differences were detected (data not shown). Third, IHC was used to assess HER2 protein overexpression without validation by FISH analysis in this study. Based on previous validation results from the same pathology laboratory, 7.4% of breast cancers with *HER2* gene amplification in FISH analysis were falsely negative by 10H8-IHC (scored as 0 or 1+) 44. If these results hold true for the current study, we could have underestimated the negative association between the recreational activity and HER2–breast cancer. The previous validation also showed 9.7% of breast cancers without *HER2* gene amplification in FISH analysis were falsely positive by 10H8-IHC (scored as 2+ or 3+); this could have led to a bias toward the null if a positive association truly exists between recreational physical activity and HER2+ breast cancer risk. Fourth, due to funding limitations, we evaluated p53 protein expression, but not *p*53 mutations. Although previous research shows that p53 protein expression and *p*53 mutation status determined by FISH analysis are strongly correlated 46, our assessment of p53 protein expression by IHC may have misclassified some tumors, which could have masked potential effect modification by p53 status in analyses of the association of recreational physical activity and breast cancer risk. Fifth, that recreational physical activity was not associated with HER2+ subtype could have been due to a lack of statistical power, as HER2+ occurs less frequently than HER2–subtype (in our study, *n* = 216 HER2+ vs. *n* = 979 HER2−). However, the majority of our risk estimates of HER2+ subtype associated with recreational physical activity are above 1 and it is plausible that the lack of an inverse relationship is real. Sixth, we were unable to examine the effect of either occupational activity or household activity on breast cancer risk because of the lack of questionnaire data on these exposures. For the same reason, we did not adjust for dietary factors in our models. Seventh, our exploratory analyses assessing whether the associations of specific receptor subtypes of breast cancer with lifetime recreational physical activity vary by menopausal status showed that the overall result was defined by the result among premenopausal women as we observed no association among postmenopausal women. It is noteworthy that the number of postmenopausal women in the Women’s CARE Study was substantially lower than the number of premenopausal women due to the Study’s design. The Women’s CARE Study was restricted to women ages 35–64 years in order to focus on the impact of oral contraceptives on breast cancer risk among older premenopausal and perimenopausal women, as well as women who had menopause within the recent past few years. Hence, fewer postmenopausal women were recruited into the study, thereby limiting our ability to address whether the observed results vary by menopausal status or by HT use. Lastly, when we stratified by several tumor markers simultaneously, case numbers were small leading to insufficient statistical power rather than to Type I error. Therefore, we did not adjust *P* values for multiple testing.

In conclusion, we found that the association between recreational physical activity and risk of breast cancer varied by HER2 status. Our conclusion is based on the exploratory data from a population-based case–control study using two-sided statistical tests without correction for multiple testing. Further research will be needed to confirm that this association is limited to HER2–breast cancers and to explore possible biological mechanisms. If our findings are confirmed and biological mechanisms are elucidated, this could advance our understanding of what controls whether a tumor has a *HER2* gene mutation.

## Supporting Information

**Table S1.** Multivariable adjusted OR and 95% CI for invasive breast cancer defined by the status of each individual receptor with lifetime recreational physical activity among premenopausal women or postmenopausal women. **Table S2.** Multivariable adjusted OR and 95% CI for invasive breast cancer defined by HER2 expression level with lifetime recreational physical activity.
