# Supplementary material for: Reduced risk of breast cancer associated with recreational physical activity varies by HER2 status
Source: Cancer Med. 2015 Apr 27;4(7):1122–35. doi: 10.1002/cam4.465 (PMC4529350; doi:10.1002/cam4.465)
Supplement: Supplementary file 1 [file cam40004-1122-sd1.doc]

Supplementary Table 1. Multivariable adjusted^1^ OR and 95% CI for invasive breast cancer defined by the status of each individual receptor with lifetime recreational physical activity among premenopausal women or postmenopausal women

| Average exercise activity (annual MET h/week) | No. of participants | | |  | OR (95% CI) | | |
| --- | --- | --- | --- | --- | --- | --- | --- |
|  | Controls | Receptor negative cases | Receptor positive cases |  | Receptor negative cases vs. controls | Receptor positive cases vs. controls | Receptor negative vs. receptor positive |
| **PREMENOPAUSAL WOMEN** | |  |  |  |  |  |  |
| **Cases sub-grouped by ER status** | |  |  |  |  |  |  |
| Inactive | 187 | 66 | 69 |  | Referent | Referent | Referent |
| ≤2.2 | 158 | 34 | 53 |  | 0.64 (0.40-1.03) | 0.92 (0.60-1.42) | 0.66 (0.37-1.18) |
| 2.3-6.6 | 188 | 54 | 64 |  | 0.88 (0.57-1.35) | 0.85 (0.56-1.29) | 1.02 (0.60-1.74) |
| 6.7-15.1 | 181 | 62 | 67 |  | 0.98 (0.64-1.50) | 0.88 (0.58-1.34) | 1.07 (0.63-1.82) |
| ≥15.2 | 215 | 58 | 54 |  | 0.81 (0.52-1.24) | 0.59 (0.38-0.91) | 1.41 (0.81-2.44) |
| Trend *p*-value |  |  |  |  | *0.80* | *0.03* | *0.12* |
| Homogeneity of trends for case-control comparison | | |  |  | *P=0.12* | |  |
|  |  |  |  |  |  |  |  |
| **Cases sub-grouped by PR status** | |  |  |  |  |  |  |
| Inactive | 187 | 66 | 69 |  | Referent | Referent | Referent |
| ≤2.2 | 158 | 36 | 51 |  | 0.69 (0.43-1.11) | 0.87 (0.56-1.35) | 0.75 (0.42-1.33) |
| 2.3-6.6 | 188 | 50 | 68 |  | 0.82 (0.53-1.27) | 0.90 (0.59-1.36) | 0.88 (0.52-1.51) |
| 6.7-15.1 | 181 | 60 | 69 |  | 0.96 (0.63-1.48) | 0.90 (0.59-1.37) | 0.98 (0.58-1.67) |
| ≥15.2 | 215 | 51 | 61 |  | 0.70 (0.45-1.09) | 0.68 (0.44-1.04) | 1.03 (0.60-1.79) |
| Trend *p*-value |  |  |  |  | *0.37* | *0.13* | *0.71* |
| Homogeneity of trends for case-control comparison | | |  |  | *P=0.65* | |  |
|  |  |  |  |  |  | |  |
| **Cases sub-grouped by HER2 status** | |  |  |  |  |  |  |
| Inactive | 187 | 116 | 19 |  | Referent | Referent | Referent |
| ≤2.2 | 158 | 72 | 15 |  | 0.74 (0.51-1.07) | 1.06 (0.51-2.21) | 0.64 (0.29-1.43) |
| 2.3-6.6 | 188 | 95 | 23 |  | 0.79 (0.55-1.13) | 1.36 (0.70-2.67) | 0.58 (0.28-1.21) |
| 6.7-15.1 | 181 | 103 | 26 |  | 0.81 (0.57-1.16) | 1.71 (0.88-3.34) | 0.39 (0.19-0.80) |
| ≥15.2 | 215 | 90 | 22 |  | 0.62 (0.43-0.89) | 1.18 (0.59-2.37) | 0.43 (0.20-0.91) |
| Trend *p*-value |  |  |  |  | *0.03* | *0.34* | *0.01* |
| Homogeneity of trends for case-control comparison | | |  |  | *P=0.04* | |  |
|  |  |  |  |  |  |  |  |
| **Cases sub-grouped by p53 status** | |  |  |  |  |  |  |
| Inactive | 187 | 94 | 41 |  | Referent | Referent | Referent |
| ≤2.2 | 158 | 59 | 28 |  | 0.73 (0.49-1.09) | 0.94 (0.55-1.62) | 0.74 (0.40-1.38) |
| 2.3-6.6 | 188 | 85 | 33 |  | 0.85 (0.59-1.24) | 0.89 (0.52-1.50) | 0.94 (0.52-1.69) |
| 6.7-15.1 | 181 | 92 | 37 |  | 0.93 (0.64-1.35) | 0.91 (0.54-1.54) | 0.97 (0.55-1.73) |
| ≥15.2 | 215 | 72 | 40 |  | 0.62 (0.42-0.91) | 0.88 (0.52-1.47) | 0.64 (0.36-1.16) |
| Trend *p*-value |  |  |  |  | *0.09* | *0.63* | *0.33* |
| Homogeneity of trends for case-control comparison | | |  |  | *P=0.48* | |  |
| **POSTMENOPAUSAL WOMEN**^2^ | |  |  |  |  |  |  |
| **Cases sub-grouped by ER status** | |  |  |  |  |  |  |
| Inactive | 251 | 53 | 87 |  | Referent | Referent | Referent |
| ≤2.2 | 165 | 34 | 72 |  | 0.98 (0.60-1.60) | 1.08 (0.73-1.59) | 0.81 (0.45-1.45) |
| 2.3-6.6 | 138 | 35 | 67 |  | 1.18 (0.72-1.94) | 1.21 (0.81-1.82) | 0.94 (0.52-1.70) |
| 6.7-15.1 | 150 | 21 | 50 |  | 0.69 (0.39-1.20) | 0.81 (0.53-1.24) | 0.85 (0.44-1.68) |
| ≥15.2 | 127 | 31 | 47 |  | 1.20 (0.71-2.01) | 0.92 (0.59-1.43) | 1.30 (0.69-2.46) |
| Trend *p*-value |  |  |  |  | *0.97* | *0.42* | *0.51* |
| Homogeneity of trends for case-control comparison | | |  |  | *P=0.55* | |  |
|  |  |  |  |  |  |  |  |
| **Cases sub-grouped by PR status** | |  |  |  |  |  |  |
| Inactive | 251 | 63 | 77 |  | Referent | Referent | Referent |
| ≤2.2 | 165 | 47 | 59 |  | 1.11 (0.71-1.73) | 0.98 (0.65-1.48) | 1.05 (0.61-1.81) |
| 2.3-6.6 | 138 | 43 | 59 |  | 1.17 (0.74-1.86) | 1.20 (0.79-1.82) | 0.94 (0.54-1.65) |
| 6.7-15.1 | 150 | 27 | 44 |  | 0.68 (0.40-1.14) | 0.81 (0.52-1.27) | 0.85 (0.45-1.60) |
| ≥15.2 | 127 | 34 | 44 |  | 1.04 (0.64-1.71) | 0.97 (0.62-1.53) | 1.09 (0.59-2.02) |
| Trend *p*-value |  |  |  |  | *0.55* | *0.67* | *0.96* |
| Homogeneity of trends for case-control comparison | | |  |  | *P=0.58* | |  |
|  |  |  |  |  |  | |  |
| **Cases sub-grouped by HER2 status** | |  |  |  |  |  |  |
| Inactive | 251 | 114 | 26 |  | Referent | Referent | Referent |
| ≤2.2 | 165 | 82 | 24 |  | 0.95 (0.66-1.36) | 1.47 (0.80-2.71) | 0.73 (0.38-1.42) |
| 2.3-6.6 | 138 | 80 | 22 |  | 1.13 (0.78-1.63) | 1.48 (0.79-2.80) | 0.84 (0.42-1.67) |
| 6.7-15.1 | 150 | 57 | 14 |  | 0.73 (0.49-1.08) | 0.87 (0.43-1.77) | 0.97 (0.44-2.13) |
| ≥15.2 | 127 | 67 | 11 |  | 1.05 (0.71-1.55) | 0.79 (0.37-1.70) | 1.64 (0.71-3.80) |
| Trend *p*-value |  |  |  |  | *0.69* | *0.40* | *0.29* |
| Homogeneity of trends for case-control comparison | | |  |  | *P=0.56* | |  |
|  |  |  |  |  |  |  |  |
| **Cases sub-grouped by p53 status** | |  |  |  |  |  |  |
| Inactive | 251 | 99 | 41 |  | Referent | Referent | Referent |
| ≤2.2 | 165 | 81 | 25 |  | 1.09 (0.76-1.58) | 0.92 (0.53-1.60) | 1.26 (0.68-2.34) |
| 2.3-6.6 | 138 | 79 | 23 |  | 1.32 (0.90-1.93) | 0.90 (0.51-1.61) | 1.44 (0.76-2.74) |
| 6.7-15.1 | 150 | 58 | 13 |  | 0.88 (0.59-1.31) | 0.47 (0.24-0.92) | 1.99 (0.93-4.25) |
| ≥15.2 | 127 | 60 | 18 |  | 1.10 (0.73-1.65) | 0.77 (0.41-1.44) | 1.52 (0.75-3.11) |
| Trend *p*-value |  |  |  |  | *0.97* | *0.11* | *0.10* |
| Homogeneity of trends for case-control comparison | | |  |  | *P=0.13* | |  |

^1^Adjusted for study site, race, education, age, family history of breast cancer, age at menarche, parity, body mass index, and the duration of oral contraceptive use. ^2^Additionally adjusted for hormone therapy use. Abbreviations: OR, odds ratio; CI, confidence interval. ER, estrogen receptor; PR, progesterone receptor; HER2, human epidermal growth factor receptor 2.

Supplementary Table 2. Multivariable adjusted^1^ OR and 95% CI for invasive breast cancer defined by HER2 expression level with lifetime recreational physical activity

| Average exercise activity (annual MET h/week) | No. of controls | HER2 expression level | | | | | |
| --- | --- | --- | --- | --- | --- | --- | --- |
|  |  | Negative/weakly positive | | Moderately positive | | Strongly positive | |
|  |  | No. of cases | OR (95% CI) | No. of cases | OR (95% CI) | No. of cases | OR (95% CI) |
| **Overall** |  |  |  |  |  |  |  |
| Inactive | 500 | 258 | Referent | 36 | Referent | 13 | Referent |
| ≤2.2 | 373 | 169 | 0.83 (0.65-1.06) | 27 | 1.05 (0.62-1.78) | 12 | 1.39 (0.62-3.14) |
| 2.3-6.6 | 369 | 201 | 0.96 (0.75-1.21) | 35 | 1.33 (0.80-2.20) | 14 | 1.48 (0.67-3.28) |
| 6.7-15.1 | 374 | 173 | 0.77 (0.60-0.99) | 24 | 0.92 (0.52-1.60) | 18 | 1.90 (0.88-4.06) |
| ≥15.2 | 396 | 178 | 0.77 (0.60-0.99) | 28 | 1.01 (0.59-1.74) | 9 | 0.89 (0.36-2.17) |
| Trend *p*-value |  |  | *0.04* |  | *0.91* |  | *0.76* |
| Homogeneity of trends | |  | *P=0.48* | | | | |
|  |  |  |  |  |  |  |  |
| **Restricted to women without first-degree family history** | | | |  |  |  |  |
| Inactive | 453 | 219 | Referent | 30 | Referent | 12 | Referent |
| ≤2.2 | 338 | 143 | 0.85 (0.65-1.10) | 23 | 1.08 (0.61-1.92) | 9 | 1.13 (0.46-2.74) |
| 2.3-6.6 | 339 | 173 | 0.96 (0.74-1.23) | 32 | 1.43 (0.83-2.45) | 13 | 1.40 (0.61-3.19) |
| 6.7-15.1 | 338 | 141 | 0.76 (0.58-0.99) | 21 | 0.95 (0.52-1.73) | 16 | 1.70 (0.76-3.79) |
| ≥15.2 | 379 | 147 | 0.72 (0.55-0.93) | 21 | 0.83 (0.46-1.53) | 9 | 0.89 (0.36-2.21) |
| Trend *p*-value |  |  | *0.01* |  | *0.53* |  | *0.77* |
| Homogeneity of trends | |  | *P=0.50* | | | | |

^1^Adjusted for study site, race, education, age, family history of breast cancer, age at menarche, parity, a four-category variable combining menopausal status and hormone therapy use, body mass index, and the duration of oral contraceptive use. Abbreviations: OR, odds ratio; CI, confidence interval. HER2, human epidermal growth factor receptor 2.
